# Chicago Pathological Society

**Published:** 1888-09

**Authors:** 


### Society Reports.

### CHICAGO PATHOLOGICAL SOCIETY.

STATED MEETING, August 6th.

President, Professor I. N. Danforth, in the chair.

The session was opened by Dr. H. N. Moyer, who presented a specimen, and said: I have here a brain, taken late this afternoon from a man who died at the Temporary Detention Hospital at 8 a. m. Unfortunately, I have no history of the case, as the man died two hours after he was admitted, and we could obtain no account of his condition previous to admission. When brought in, he was in a condition of acute delirium. He was noisy; threw his arms about furiously. Hallucinations rapidly succeeded one another and he died in this condition from failure of the heart, and without symptoms of coma or compression of the brain. A diagnosis is, of course, impossible in the absence of a history of the case; but the symptoms, so far as they were observed, resemble somewhat *delirium grave* as defined by Spitzka.

The *post mortem* examination revealed intense congestion of the membrane of the brane, as may be seen by examining the specimen. The injection is arterial; indeed, the veins contain less blood than usual; the larger sinuses are almost empty. The brain is normal in consistency and unusually dry. The ventricles are quite empty and the subarachnoidian space contains no fluid. As the membrane is stripped from the convolutions they are seen to be of a pale rosy tint. The *puncta vasculosa* are larger and more numerous.

The abdominal and thoracic organs presented nothing abnormal, with the exception of the liver, which was infiltrated with fat. This suggests that possibly the man was in the last stage of delirium tremens, a theory not inconsistent with the *post mortem* findings.

Dr. J. D. Skeer: I have examined a great many brains, but have never seen the convolutions reddened to such an extent as in this case.

Professor H. M. Lyman: I once had the opportunity of seeing a parallel case to this, in which I had the advantage of the history. I am very sorry we have not got the history of Dr. Moyer's interesting case. The case to which I have reference was that of a young girl, about 15 years of age, who had been attacked with scarlet fever. She had been an epileptic patient and had had a number of epileptic convulsions prior to the attack of scarlet fever. She died in the acute stage before the eruption had disappeared—about the fifth day of the disease—in a state of coma following delirium.

The *post mortem* examination revealed exactly the condition we have here—that intense congestion of the meninges; that rosy appearance of the cortical brain matter and general dryness of the tissues, not merely confined to the brain, but diffused through the body, such as we find in typhoid fever and that class of cases; and I suspect we have here a case of intoxication of some kind as a cause of the acute meningitis.

Dr. Skeer: When a brain is removed from the calvarium and allowed to be exposed to the air ten or twelve hours, the pia mater will assume a reddened appearance, precisely like the specimen exhibited.

Dr. Lydston : The suggestion has been made that the appearances in this case are somewhat characteristic of toxic infection of the brain and are also characteristic of certain cases of acute delirium. This recalls to me several cases of acute and violent delirium, apparently due to acute and violent meningitis, in which there were no pathological changes evident *post mortem*. One of these was a case of erysipelas of the orbit, complicated by delirium tremens in which violent sthenic delirium developed, during the course of which the woman jumped through a window, in an attempt to escape from the hospital, carrying sash and all with her. The other was a case of cellulitis of the neck, evidently due to some septic infection in which the patient developed violent acute delirium, succeeded by coma and death within 24 hours after his admission into the hospital. In neither of these cases were any pathological changes evident in the brain *post mortem*. Indeed, the brain was exceptionally healthy in each case, with the exception perhaps of a trifling venous congestion which might occur in almost any case from apparently slight causes. I attributed the cerebral symptoms in these cases to the impressions made upon the cerebral tissues by the intense toxic infection present. There seems to be an especial tendency to cerebral symptoms when the septic process exists in structures contiguous to the head, and this tendency is not explicable entirely upon the theory of acute congestion or hyperæmia, although these conditions may enter into the causation of the cerebral trouble and present comparatively little evidence of pathological change upon *post mortem*.

Professor Lyman, replying to Dr. Lydston, said he meant alcoholic intoxication and not a condition brought about by the introduction of septic material. In case of erysipelatous disease, such as Dr. Lydston alludes to, he would be inclined to refer the cerebral symptoms to the effects of the poison generated by the microbes in the erysipelatous tissues, the ptomaine being absorbed and diffused through the tissues, acting as a poison upon the nerve centres, death being produced by toxicæmia in that way.

The next thing in order was a discussion on Dr. Strong's papaper, "Meat Diet as a Cause of Calculi."

Dr. T. D. Fitch : I believe it is the supposition that all albuminous substances pass through uric acid before being excreted, as is stated by the authors I have read, and is generally accepted by the profession. There are other causes that act quite as frequently and forcibly in the production or increase of uric acid as that of meat-eating, or the eating of nitrogenous food, and while I have been thinking about this subject more or less, I have met a good many hearty, hale men of advanced years, have inquired of them how often they ate meat and how much they ate, and the majority I have talked with say they have never suffered from rheumatism gravel or gout, although they have eaten meat two or three times a day most of the time during their lives. The authors I have read attribute the excessive amount of uric acid to nervous influences, disturbances of the functions of the liver and to other organs in the system quite as frequently as that of excessive meat eating. They commend fish and white meat of fowls as a good article of diet in persons suffering from gout, rheumatism, or any diseases arising from excessive uric acid.

We all know fish contains almost exclusively nitrogenous, elements, that there are only some traces of carbonaceous material in fish, there being 17 per centum of nitrogenous material in the meat of fish.

The white meat of chicken and fowls is nearly all nitrogenous and we must remember also that there are a large number of the vegetable articles of diet which we use that contain large proportions of nitrogenous material, corn and rice, for instance. An investigation, which was made during the siege of Paris by a prominent physician (Sethby), showed the smallest amount and due proportion of nutriment that soldiers and people could live upon during the siege, or at any time, and the proportion of nitrogenous and carbonaceous material which should exist in the food to give healthy nutrition. There was in idleness one part of nitrogenous to 7 parts of carbonaceous, or to be more exact, two ounces and 67 hundredths of nitrogenous food to 19 ounces and 61 hundredths of carbonaceous. In ordinary labor, one nitrogenous to 6½ carbonaceous; in hard labor, 5 ounces and 81 hundredths of nitrogenous, to 34 ounces and 97 hundredths of carbonaceous. Now corn is the only substance giving due proportion of the two elements, viz., 1 to 6½ for ordinary labor.

In children, who are fed mostly upon milk, which is highly carbonaceous, having but a small percentage of nitrogen in it, the proportion is one of nitrogenous to 11 of carbonaceous. If children are fed upon carbonaceous food, the carbonaceous elements highly predominating, why is it we have such a large percentage of calculi?

I do not remember the name of the author who gives the statistics of thousands of cases of calculi mentioned in Ziemssen; 45

per centum occurred in children. If children are fed upon a carbonaceous article of diet principally, what influence can nitrogenous food have in producing calculi in them ? It seems to me the importance of this matter is overestimated.

Professor Parkes states that on withdrawing nitrogenous food from patients suffering from any of these diseases, gout, rheumatism, scorbutus or lithæmia, there was no apparent lessening of the amount of uric acid in the blood, urine or tissues ; consequently he was inclined to think that its office in producing excessive uric acid in the blood was greatly exaggerated.

The President: We are in the midst of the uric acid cycle, and especially with reference to meat-eating. Nevertheless, I do believe that meat diet tends to the production of urinary calculi under certain conditions:

1. If one consumes an inordinate amount of meat, more than the functions of the body demand, I think that fact alone tends to its production; but more so if that is accompanied with a sedentary life. Unless the meat-eater is engaged in a daily avocation where he gets a large amount of muscular exercise, so that there is a demand for the flesh diet, I think that that tends to aid in the production of lithic acid excess.

2. If this consumption of meat and sedentary life are accompanied with an inordinate use of intoxicants—alcohol in any form, wine, whiskey, beer or otherwise—I believe it acts also as an additional factor in its production.

3. If we add to that the influence of heredity, which may mean a rheumatic or gout ancestry, the likelihood is very great that calculi in the kidney may develop.

But while we always expect under such circumstances to get lithic acid calculi, it does not follow that we are *always* going to get them; for we sometimes get oxalic and phosphatic calculi and sometimes mixed calculi, but we are more likely to get lithic acid calculi in the majority of cases. If along with these conditions there be a faulty condition of the liver, or any form of chronic hepatic disease, it serves as an additional aid towards the production of lithic acid calculi; for we are nowadays told that the function of the liver is not by any means altogether that of secreting bile, but has largely to do with the metabolic changes which occur in the tissues; that waste materials must pass through the liver and subject themselves to the modifying power, so that refuse material from the higher structures of the body must first become lithic acid and then urea, in order to escape by way of the kidneys in the most favorable way. If there be any interruption of function of the liver through organic disease, a diabetic condition, or from the inordinate use of stimulants, this change from lithic acid to urea does not occur completely; consequently there accumulates in the blood lithic acid which the kidneys cannot eliminate; hence it is precipitated in the renal tubules, and this commences the formation of a calculus.

It takes something more than a meat diet, three times a day, to produce lithæmia in most people. It may occur in persons with active habits of life, abstinence, active liver, etc. But I have never met with a case where it has occurred without some adjunct beyond and above merely the use of meat.

Professor Danforth, at the close of his remarks, exhibited several specimens of calculi to the society.

One phial contained 75 to 100 calculi which were passed by a patient at one urination.

Professor C. C. P. Silva: When I first started out to practice on the coast of Spain, in a small country town, where the inhabitants (mostly laboring class) did not eat meat more than probably twice a week, and lived almost exclusively on vegetables, plenty of olives and cheese, milk, etc., where wine was not used very profusely, for they could not afford it, I saw many cases of stone and gravel, both in male and female. I remember one very extraordinary case of the passing of a stone, by a woman, the size and shape of an ordinary pear. The stone was passed from the bladder spontaneously, with excessive pain, and the woman, who was pregnant at the time, supposed the pain was labor, but *true* labor only occurred a month afterwards. With this case, I saw, without exaggeration, in a population of 5,000 inhabitants, during the seven years I practiced there, 100 cases of gravel and stone, of all forms, sizes and shapes; so that there is something else besides meat-eating that produces calculi. It probably depends upon some climatic conditions, or the condition of the water. It is not dependent upon food or drink alone. I find that these conditions of concretions of the bladder are found in certain localities circumscribed to small areas. What causes it I do not know. It is claimed that water being too calcareous will help in producing it. I believe the excessive use of acids will produce precipitation of uric acid and form a nucleus for the formation of stone. In those to which I allude, the people use considerable lemons; they are cheap; they cost nothing; all you have to do is to pick them off of a tree; so that it is claimed that the excessive use of lemons will produce precipitation of uric acid and forms a nucleus for the formation of stone.

Dr. William E. Clark: Do you think that is well established—the vegetable acid theory?

Professor Silva: It is received as true.

Dr. J. R. McCullough had eaten meat since he was 10 or 12 years of age two or three times a day, and occasionally drank stimulants; he had never had any calculi or trouble with the kidneys.

Dr. J. J. M. Angear: In our efforts to find the prime cause of renal calculi, we must bear in mind some fundamental principles.

The materials of which the calculi are composed are normal constituents or products of the body. The cause must be within and not without the body. These materials are held in solution in the healthy body and normal environments.

The cause of calculi is not the introduction of the material into the body as food and drink, but it is the cause of the material being precipitated or crystallized, and thereby not eliminated. The cause of the calculi and the cause of crystallization is one and the same thing. The *mere* increase of the quantity of the material in the body, or an accumulation of it in the urine as a cause of calculi, is an exploded idea.

Uric acid at the temperature of the body, with normal environments, is soluble. The sediment—the red brick-dust—is crystals of uric acid, which form after the urine has left the body. The urine at the time it leaves the body is clear and transparent; if not, there is an abnormal condition, not merely a superabundance of uric acid.

If we eat excessively of anything, the major portion of the surplus passes off through the alimentary canal without entering the system. I doubt very much whether such enormous quantities of meat, which I see eaten at our restaurants, more than enough for three ordinary persons, is digested. It simply remains meat and is thrown out of the alimentary canal as such. Without having made any experiments to verify what I say, I am inclined to think that had we the ability and disposition to analyze the fecal matter of such persons, we should find a large amount of meat in the feces, and comparatively but few of these people are annoyed with rheumatism or troubled with the gout.

The cause of renal and vesical calculi, except in cases of the introduction of foreign bodies into the bladder, is one and the same. The cause is to be looked for in the kidney as the location, and the cause of the crystallization as the prime cause of renal calculi.

Foreign matter in a solution of crystallizable material causes or hastens crystallization. Dead matter is foreign matter in the animal economy. That which devitalizes the renal cells, coagulates mucus, produces pus, causes crystallization of the solid matter in the urine, and this takes place in the tubuli uriniferi, or, in other words, causes renal calculi.

Some authors are of the opinion that there is a specific ferment, or that the renal cells throw out an abnormal colloid material which tends to precipitate the solids of the urine.

As far as gout is concerned, which has been alluded to, it is not free uric acid, nor is it the urates alone, but we have the phosphate of lime deposited in the cartilages of some of our gouty patients. We read and hear of the English nabobs living upon roast beef and drinking brandy, brandy being the favorite English drink. The meat may cause the uric acid, but not the phosphate of lime. Lime enters largely into the composition of the gouty deposits. The gouty material is precipitated from the blood and not from the urine, and perhaps the kidneys "have nothing to do with the case."

In my opinion, when you have found the cause of the precipitation in the kidney, you have found the cause of calculi, and not till then.

Dr. G. Frank Lydston: The question of diet in the etiology of calculous affection is of considerable importance, but I believe that the tendency has been toward an exaggerated estimate of the evils resulting from various alleged unphysiological forms of diet rather than the contrary.

In the case of children, it must be acknowledged that in a large proportion of instances there is something more than the ingestion of an excessive amount of meat or other nitrogenized elements of food necessary to the production of urinary calculi. Children, as is well known, and particularly infants, seem to present an especial predisposition towards lithic conditions of the urine. *Why this is* we cannot say. It certainly is not because the majority of children are fed an excessive amount of nitrogenous aliment, for quite a proportion of cases of calculus in children originate prior to the period when a meat diet is ordinarily begun. Thus the statistics of Thompson show that out of 1,800 cases of calculus, taken at random, something over 500 occurred in children under 5 years of age, and it is hardly reasonable to suppose that a large majority of those children had been fed an excessive quantity of nitrogenized matters. Indeed, most of the cases occur among the poorer classes, to whom meat is a luxury, and rarely indulged in to excess. The more favored class of meat-eating children seldom have calculus. Calculus is slow in its formation, and is almost an inevitable consequence; it may be inferred that a large proportion of the cases which occur in children began long before the assumption of a meat diet.

Cases of congenital calculus, while they are very rare, have been observed. It is barely possible that the marked tendency to lithiasis in children, and the occurrence of congenital calculi, are applicable upon the theory of an excessive ingestion of nitrogenous material, and in some cases to liquors by the mothers of the affected children.

Faulty diet and intemperance upon the part of the mother, hereditary cachexia, struma, unhygienic surroundings, perverted quality and insufficient quantity of food, and lack of proper exercise in open air, in short, all the well-known causes of mal-assimilation in children, bear a much more important relation to the etiology of calculus than does the simple ingestion of excessive quantities of meat, which must necessarily be but a secondary consideration and really a minor factor of the great whole which comprises the etiology of calculus in children.

Calculus seems to have a special predilection for the two extremes of life, being due in the very young to the conditions just enumerated, and in advance life to intemperance in eating and drinking, hereditary tendencies and the various obstructive diseases of the genito-urinary tract.

There is one consideration which seems to have been forgotten by our surgical authorities in connection with this subject, and that is the difference which exists between the functions of the infantile and those of the adult liver. Precisely what relation the peculiar arrangement of the liver in young children during intrauterine life has to the formation of vesical calculus remains for the physiologist to determine.

After all that has been said upon the etiology of calculus, there still remains something which we are unable to explain in the way of differences in the relative predisposition of the sexes, the frequency of calculus in some locations as compared with others and the varying predisposition of different races. Although these circumstances have been explained by different authorities upon the basis of variation in the composition of food and drink and in the habits of the residents of different localities, there still, in my estimation, remain etiological questions which have not yet been satisfactorily answered.

Dr. Lydston then read a paper on "The Evolution of Chancroid and Gonorrhœa, and the relation of evolution to infectious diseases in general."

He said chancroid was formerly supposed to be identical with syphilis, and the term "chancre" was applied indiscriminately to all sores of an ulcerative or indurated character which appeared upon the male or female genital organs. According to John Hunter, there was but one venereal disease, and this decidedly complex affection included the diseases which we now term gonorrhœa, chancroid and syphilis. Then came Ricord, who demonstrated the existence of two distinct diseases, namely, gonorrhœa and syphilis, but he failed to appreciate any difference between the sore which we now know to be local and non-constitutional and the sore which is always a manifestation of a general and infectious disease.

Fournier says, "Chancroid is a specific malady consisting in a peculiar ulcer, which secretes a virulent auto-inoculable pus. It is exclusively local and never gives rise to constitutional symptoms." Dr. Lydston considered this definition an excellent one.

When Drs. Taylor and Bumstead, in their excellent work upon the venereal diseases, advocated the doctrine that the chancroidal virus was not a specific entity, and that chancroidal ulcerations differed in degree only, rather than kind, from ulcers of a simple character, there were very few, indeed, who did not antagonize their views. It is only within a very short time that he had been convinced from practical experience that chancroid is a disease which may arise *de novo*, and which, in the true sense of the term, is not specific.

The conditions modifying the results of germ infection were, as he understood them, as follows:

1. The degree of virulency and vitality of the germ at the time it enters the tissues or blood of a human being.

2. The inherent vitality of the individual or his resisting power at the time of infection.

3. Individual susceptibility to the particular disease represented by the germ—i. e., idiosyncrasy.

4. The condition of the eliminative apparatus of the person affected.

5. If the disease germ has a special predilection for any particular tissue, the result will be modified by the condition of that tissue at the time of its infection, e. g., the typhoid bacillus and the coma bacillus of Cholera Asiatica, most readily affects those who have morbid conditions of the alimentary canal

Diphtheria is most apt to attack persons with acute or chronic naso-pharyngeal disease.

6. And one of the most important of all, the number of germs and the length of time during which the patient is exposed to their influence.

Although not usually attributed to evolutionary laws, some of these ideas in relation to the development of infectious diseases are already pervading the profession to a slight extent. Probably the nearest approach to a thorough exposition of the subject is an essay by Dr. DeGorrequer Griffith, of London, entitled the "Unity of Poison." In this article the learned author shows plainly the co-relation of certain infectious diseases formerly supposed to be separate and distinct affections.

The idea that the chancroidal poison is one which has always been inseparable from the human species is, of course, untenable. Somewhere along the line of ancestry, chancroid appeared, but at what time, history does not tell us. The human race in general must have begun with a considerable capital in the form of a healthy organization, and every disease which now affects unfortunate humanity must necessarily have developed since the species originated. As the races have become differentiated, or have diverged, new circumstances of environment have been encountered which have modified the organism of the human being, and in the course of evolutionary progression many and various diseases have arisen.

This fact has been due to:

1st. The appearance upon the scene of weaker and more susceptible organizations than those of the parent stock.

2d. Changes of telluric and climatic influences.

3rd. Injuries and vicissitudes experienced in the struggle for existence, modifying the organisms of numerous individuals and such modification being transmitted to their descendants.

4th. Varying character and quantity of food and drink, alcoholics within a considerable number of generations, having exerted a marked influence.

5th. Varying sanitary circumstances, involving crowd poison and other forms of noxious and contaminating animal matter.

6th. Varying personal hygiene, involving cleanliness, exposure to cold and wet and other influences which may modify individual constitutions. The question of sexual habits here enters into consideration and is of especial importance in its bearing upon the evolution of venereal disease.

7th. The gradual and certain evolution and differentiation of and acquirement of new properties by living germs.

Gonorrhœa and chancroid have arisen in a manner precisely similar to the evolution of other infectious diseases, and while it is premature to say that the poisons of the two diseases are precisely identical, he is firmly convinced that they are different in degree, rather than kind and of a similar origin, to say the least.

As the circumstances of uncleanliness, unhealthy secretions, local irritation, heat, moisture and deprivation of free air and light, favor the development of germs, and particularly those of decomposition, it may be readily understood that after a time such a bacterial development actually takes place in the vaginæ of some women. The innocuous germs of the atmosphere enter and begin their work of procreation of multiplication, decomposition occurs and *pari passu* with it new germs appear upon the scene which differ from the parent stock, and so the process goes on until a very irritating poison is developed. If during this time the discharge from a diseased urethra be added to the noxious materials, or if semen be deposited in this "hot bed of putrefaction," so much the better for the development of a "specific" poison.

As to the conditions which modify the results of the virus generated *de novo* in the human vagina, these are as follows:

1. It is obvious that much depends upon (a) the age of the decomposition; (b) the degree of inflammation present; (c) the frequency of coitus; (d) the constitution and habits of woman; (e) the character of any semen or urethral discharges which may be deposited in the vagina; (f) the degree of cleanliness of the woman.

2. The amount and degree of virulency of the virus deposited upon the absorbent surface in another individual.

3. The cleanliness, local and constitutional condition, habits and sexual hygiene of the recipient of the cultivating virus.

4. Individual predisposition.

